# Poster 1021: Use of paper filter discs for measurement of local biomarkers of nasal mucosal inflammation

**DOI:** 10.1186/1939-4551-7-S1-P12

**Published:** 2014-02-03

**Authors:** Margot Berings, Lien Calus, Lien Devuyst, Thibaut Van Zele, Natalie De Ruyck, Claus Bachert, Philippe Gevaert

**Affiliations:** 1 Upper Airway Research Laboratory, Ghent University, Ghent, Belgium; 2 Division of ENT Diseases, Clintec, Karolinska Institutet, Stockholm, Sweden

## Background

Allergic rhinitis has been characterized by an important local mucosal inflammation. Therefore we aimed to measure mucosal immunoglobulins and tryptase in nasal secretions by means of paper filter discs in allergic rhinitis (AR) patients in comparison to controls. The purpose of this study was to evaluate the suitability of this approach for use in future clinical trials.

## Methods

Nasal secretions from 12 patients with AR to grass pollen and 12 healthy controls were collected. Two pre-weighed filter discs were placed bilaterally under direct visualization on the anterior third of the nasal septum. Five minutes later, they were removed and weighted again. After adding 2 ml 0,9% NaCl solution to the discs and centrifugation, the nasal secretions were stored in aliquots at -20°C until analysis. Biomarkers such as total IgE, grass pollen specific (gx3) IgE, total IgG, IgG_4_ and tryptase were measured. Patients with AR were selected based on symptoms during the grass pollen season and based on a positive skin prick test for grass pollen. Control patients were healthy patients without rhinological or allergic disease and with negative skin prick test to the standard panel of allergens.

## Results

In nasal secretions of AR patients, significant higher levels of total IgE (43.13 kU/l [2.83-75.36] vs 1.66 kU/l [1.66-3.21] resp., P=0.006), grass pollen specific IgE (14.11 kU/l [2.30-24.56] vs 1.66 kU/l [1.66-1.66] resp., P=0.001) and tryptase (33.67 μg/l [8.28-85.07] vs 8.28 μg/l [8.28-8.28], P=0.032) were found compared to controls. Total IgG levels tend to be lower in AR patients compared to controls (182.62 mg/l [86.04-314.56] vs 256.17 mg/l [192.67-457.25], P=0.288) and the levels of IgG4 were rather similar in both groups (12.87 mg/l [4.79-53.48] vs 16.75 mg/dl [3.36-33.26], P=0.833).

## Conclusions

We were able to measure different biomarkers of nasal mucosal inflammation in nasal secretions collected by means of paper filter discs. As this approach is considerably less invasive and requires less expertise than nasal biopsy, and as –in this small sample– patients with AR had significant higher levels of total IgE, specific IgE and tryptase compared to healthy controls, this method seems to be suitable for monitoring local mucosal inflammation in allergic rhinitis.

